# Dementia: Merging frontiers and emerging vistas

**Published:** 2009-01

**Authors:** T. S. Sathyanarayana Rao, M. R. Asha

**Affiliations:** Department of Psychiatry, JSS University, JSS Medical College, Mysore - 570004, India; 1 Reiki Practitioner & Freelance writer, 788/160, 18^th^ Cross, Ramanuja Road, Mysore, India

If the head and body are to be well,

You need to begin by curing the soul.

**Plato, *Dialogues***

All emotional illness is basically unfinished business and unexpressed truth. For, truth has the power to heal, protect, guide, and inspire. The cardinal principle of psychotherapy is to help the patient become (more) truthful about his/her feelings. A psychotherapist may be elegantly described as a choreographer of emotions. A healthy and wise approach to mental illness would be the one based on honesty and truthful living which has a tremendous therapeutic impact. This preventive and healing aspect of natural therapy has been unfortunately obscured and almost drowned in the drone of (sometimes) premature pharmacological interventions. When we are dealing with potentially irreversible disorders, *prevention and delaying the onset* gain enormous significance than combating after the onset. Natural therapy might be one of the best secrets of preventing emotional illness including dementia which is very often irreversible. It is the general lack of holistic approach in clinical practice that frustrates the patient and the caregivers as well, because mental illness is no longer viewed as an isolated case of neuropathological disarray. It has biological, psychological, emotional, social, economic, cultural, ethical, AND, spiritual dimensions, whose silent cries have been largely ignored thus far. Now, the paradigm is undergoing a welcome change (slowly but surely) with neuroscientists working in harmony with spiritualists evidenced by the emergence of Neurotheology.

Manifesting as a set of symptoms representing a progressive decline in cognitive processes, dementia derives its name from the Latin root “*de*” meaning “apart or away” and “*mens*” (genitive- *mentis*) meaning “mind”. A lonely sojourn of utter confusion and frustration for the patient, dementia has become a huge global problem.

Paranoid orientations tend to develop in demented people who have premorbid sensitive and suspicious nature. Existing personality traits are intensified by degenerative brain biochemistry and also the stress accompanying old age. It has been observed that elderly people with Alzheimer’s disease (AD) are usually unaware of their own cognitive deficits and hence do not attribute negative incidents to this cause.[1]

The sense of rejection, abandonment, helplessness and the deepening urgency to grab the fragments of slipping self-identity and self-worth are nearly palpable in many dementia patients. Minds which had once been beautiful, active and vibrantly alive gradually become blank and disconnected struggling endlessly and painfully to make sense of disjointed images. The shell of the mind closes in on its empty self.

Dementia results in three principal kinds of loss: i. the loss of love, ii. the loss of control – intertwined with fear of rejection and making the person more vulnerable to loss of love, and iii. the loss of self esteem – including being embarrassed and being exposed as flawed.[2]

The magnitude of this disease is straining family and societal resources. The often associated problems of marked agitation, uncontrolled eating habits, inappropriate sexual behavior, senselessly repetitive behavior, unpredictable emotional outbursts, hoarding of useless objects, etc. in demented people can be enormously distressing to the caregivers, especially in the long run.

AD patients with depression tend to develop extremely morbid preoccupations and delusions – hypochondriacal ideas about suffering from various dreadful diseases, for instance, often viewed as punishments for past sins. As a way of atonement, suicide is a possibility in such cases.

Robertson (1982) aptly stated that “meaningful life can be prolonged if brain degeneration could be held at bay. But in broad terms, reasonable cerebral function is the key to the quality of survival in the elderly”.[3]

AD appears to be the product of not a single or even a limited array of specific causal factors. Often it is the consequence of an accumulation of risk factors – both environmental and genetic – that eventually exceed some clinical threshold when disease manifests.

As the disease advances, caregivers are confronted not only with challenging management problems, but also with the “social death” of the patient as a person.[4] It is well recognized that, as a group, caregivers are at extraordinarily high risk for depression.[5] The progressive marked confusion, disorientation for person, place and time, gross and argumentative behavior, possible sudden eruption of combative violence in demented people can place an unendurable strain on caregivers.

The time has now come, as a means of shared humanity, to break the boundaries of ordinary reasoning and expand our empathic understanding of the essence of the person with dementia. It begins with unconditional acceptance, modulating the expectations, balancing and optimizing the influences of and interactions with the patient’s internal and external environment. *Living in the present* is perhaps all that a person with dementia can really experience as the condition advances, when past and future hold no meaning for him/her. Not compromising their basic dignity and not denying their value as humans, they need to be nurtured, empowered to respect themselves and encouraged to preserve whatever remains of their cognitive functions.

Claire Biernacki states that while interpersonal care based on “Person centered model” is the order of the day which is likely to improve the quality of the patient’s day–today life, it is equally vital to maintain our awareness of advances in medical field that would enhance the clinical practice.[6]

In the changing paradigm of enhanced awareness about consciousness, a burgeoning idea is that the challenges we face as humans on earth are no longer regarded as meaningless suffering or random occurrences. They are viewed as life lessons consciously chosen during prebirth planning by our souls in order to evolve.[7] Albeit appearing a revolutionary and mind boggling concept, a careful consideration of and pondering the same would be immensely beneficial to demented people (as long as they can think) and equally or more to their caregivers in replacing feelings of fear, resentment, self-pity, blame and anger by a focus on growth, accompanied by new understanding and clarity. This gift of understanding would go a long way in preventing depression in both the groups.

Emerging vistas indicate that the conventional medical world is on the threshold of recognizing the link between energy or spiritual dysfunction and illness. That survival strategies are passed along in our DNA has been shown by Evolutionary psychologists.[8] Caroline Myss points to an intriguing concept that depression and even physical illness may result from a failure to discover our purpose in life – the sacred contract – that we and only we can fulfill.[9] Larry Dossey writes in “Meaning and Medicine” that there is an increasing need to practice “Era III Medicine” – therapies that combine spiritual, physical and holistic approaches to physical and emotional healing.[10] Neurobiologist Candace Pert has proved that neuropeptides– the chemicals triggered by emotions– are actually thoughts transformed into matter. Our emotions physically stay in our bodies and interact with our cells. The emotional biochemistry in the brain is what ultimately becomes our biology.[11] Her research has provided the evidence of the biochemical basis for awareness and consciousness. In the recent years, complementary and alternative medicine including dietary interventions and aromatherapy have revolutionized how the medical community views health and disease. Although the term “Energy Medicine” is making inroads to medical psyche in the recent years, many medical technologies have been using different forms of energy for diagnosis and treatment – X–rays and of late, magnetic resonance imaging (MRI), falling into this category.

Now it has been established that the body and its immune system possess tremendous healing potential. According to Dr. Zhi Gang Sha, “Lack of sufficient energy” is the root cause of degenerative diseases such as Alzheimer’s. The suggested healing remedy is to increase the energy density around the cells.[12]

Although people with dementia are generally regarded as having lost their minds, the beauty inherent in their spirit remains untainted and eternal. Statistics does not apply to human spirit – dementia or not.

Here is a befitting tribute to the power of human spirit, envisaged as “Beautiful Soul” by the second author.

## I’M A BEAUTIFUL SOUL

I’m a beautiful soul
Loving, eternal and whole

I’m a grateful smiling light
Peaceful, joyous and bright

I’m an energetic tree,
Balanced, blissful and free

I’m a lovely little flower
Blooming every hour

I’m a daring bird, soaring high
A non-judging observer, as life goes by

I’m a mighty, humble mountain
And the sprightly healing fountain

I’m a passionate sea
Willing and able to “BE”

I’m Mother earth, ecstatic
Grounded, dependable and authentic

I’m the vast sky, awesome
Centered within the humdrum

I’m a beautiful soul
Loving, eternal and whole!
